# Association between different types of lung cancer and neurodegenerative diseases: A two-sample Mendelian randomization study

**DOI:** 10.1097/MD.0000000000045048

**Published:** 2025-10-03

**Authors:** Wentao Zheng, Ying Wang, Enjiang Zhu, Rui Xu, Ling Zhu

**Affiliations:** a The First Affiliated Hospital of Dali University, Dali, Yunnan Province, China; b Department of Orthopedics II, Mile Hospital of TCM, Mile, Yunnan Province, China; c Dermatology Department, Mile Hospital of TCM, Mile, Yunnan Province, China; d School of Basic Medical Sciences, Qujing Medical College, Qujing, Yunnan Province, China.

**Keywords:** Alzheimer disease, lung cancer, Mendelian randomization, Parkinson disease

## Abstract

Previous observational studies demonstrated that lung cancer and both Alzheimer disease (AD) and Parkinson disease (PD) may be related. However, the results of different studies are still controversial, possibly due to differences in the types of lung cancer examined. To date, no research has discussed it further yet. In this study, Mendelian randomization (MR) analysis was employed to investigate the relationship between different types of lung cancer and neurodegenerative diseases. We extracted data sets related to the exposure (lung cancer) and outcomes (AD/PD) from the Integrative Epidemiology Unit open Genome-Wide Association Study project. Then, we carried out two-sample MR analyses to explore the connection between lung cancer and both AD and PD. We also conducted sensitivity analyses on positive results from the MR analysis to make sure the results were accurate. Our two-sample MR analyses revealed that lung squamous cell carcinoma (LUSC) is negatively related to AD (OR = 0.935, 95% CI [0.892–0.980], *P* = .005). According to the sensitivity and heterogeneity analyses, no heterogeneity (*P* = .223) and pleiotropy (*P* = .548) were observed in this positive result. Thus, the findings were reliable. The IVW findings suggested that LUSC and PD did not significantly correlate (OR = 0.920, 95% CI [0.778–1.089], *P* = .332). Similarly, there was no obvious connection between lung adenocarcinoma and AD (OR = 0.968, 95% CI [0.915–1.025], *P* = .270) and PD (OR = 0.981, 95% CI [0.851–1.130], *P* = .787), according to IVW results. Results of two-sample MR analyses revealed that LUSC and a decreased risk of AD may be related.

## 1. Introduction

Lung cancer is one of the most prevalent forms of cancer worldwide, which mainly includes 2 subtypes: small cell lung cancer (SCLC) and non-small cell lung cancer (NSCLC). NSCLC is the principal subtype of lung cancer, which is further divided into lung adenocarcinoma (LUAD) and lung squamous cell carcinoma (LUSC).^[1]^ The World Health Organization’s specialist cancer agency, the International Agency for Research on Cancer, released the GLOBOCAN Cancer Report in 2023. It states that there were almost 20 million new cases in 2022. Moreover, with 2.5 million new cases, lung cancer was the most prevalent cancer across the world, accounting for 12.4% of all newly diagnosed cases. With 1.8 million cases, lung cancer also led the globe in deaths that year.^[2]^ These numbers continue to rise every year due to the rising incidence of smoking and severe environmental pollution.

Two prevalent neurodegenerative disease forms are Alzheimer disease (AD) and Parkinson disease (PD).^[3]^ Although both AD and PD are degenerative diseases of the central system, differences still exist in their pathogenesis. Pathologically, AD involves the creation of intracellular neurofibrillary tangles caused by hyperphosphorylated tau protein as well as extracellular senile plaques caused by β-amyloid deposition, which leads to damage and death of nerve cells in some regions of the brain, and finally causes cognitive dysfunction in patients.^[4]^ Differently, PD is identified by the degradation of the nigrostriatal pathway, leading to the progressive apoptosis of dopaminergic neurons. As a result, low levels of dopamine and overactivity of acetylcholine may contribute to a range of symptoms, both motor and non-motor, including bradykinesia and memory impairment.^[5]^ According to a report on the Global Burden of Diseases from 1990 to 2015: approximately 29.8 million and 6.2 million people worldwide are currently affected by AD and PD, respectively.^[6]^ In brief, AD and PD are posing heavy disease burden on older adults around the world.

Previous studies have proved that, compared with healthy controls, patients with neurodegenerative diseases are less likely to suffer from cancers. For instance, Ong et al^[7]^ conducted a cohort study to investigate whether PD and cancer risk are related, encompassing 219,194 patients with PD, and findings manifested that patients with PD were negatively correlated with the occurrence of 11 kinds of malignant tumors, including lung cancer (RR = 0.75, 95% CI [0.71–0.78], *P* < .05) and esophagus cancer (RR = 0.86, 95% CI [0.82–0.91], *P* < .05). In a meta-analysis of PD and cancer risk assessment,^[8]^ researchers included 15 relevant articles and findings demonstrated that the relative risk (RR) of lung cancer in patients with PD was 0.53, compared to control patients without PD (RR = 0.53, 95% CI [0.41–0.70], *P* < .001), indicating a substantial correlation between patients with PD and decreased chance of developing lung cancer. Moreover, similar findings were obtained from subgroup analyses based on study quality, gender, and experimental design. These outcomes were also noted in AD patients. A study by Musicco et al^[9]^ concluded that, despite both AD and cancer incidence increasing with age, patients with AD actually have a lower chance of developing cancer. Additionally, in a retrospective cohort investigation on the cancer incidence of 8097 patients with AD, the Ren et al team found that lung cancer is less common in this population (HR = 0.656; 95% CI [0.494–0.871], *P* = .004).^[10]^ There is a lower chance of cancer with both diseases, which may be attributed to similarities in their clinical presentations and the neurodegenerative characteristics they share. However, most of these studies are observational, and heterogeneity in findings may be attributed to the interference of confounding variables and direction of causality. Thus, the findings of these studies are still unreliable to some extent. Thus, current research still cannot clarify whether lung cancer is causally related to AD and PD.

Mendelian randomization (MR) is a new technique for determining the causal effect and a type of instrumental variable (IV).^[11]^ Based on the strong correlation between IV (genetic variation) and exposure, this method could minimize confounding factor bias in traditional observational studies, making it possible to determine the relationship between exposures and results. In this study, we explored the association between lung cancer and 2 common neurodegenerative diseases (AD and PD), based on single nucleotide polymorphisms (SNPs)^[12]^ at the genetic level, offering a theoretical framework for future clinical research.

## 2. Methods

### 2.1. Study design

**Figure 1.**
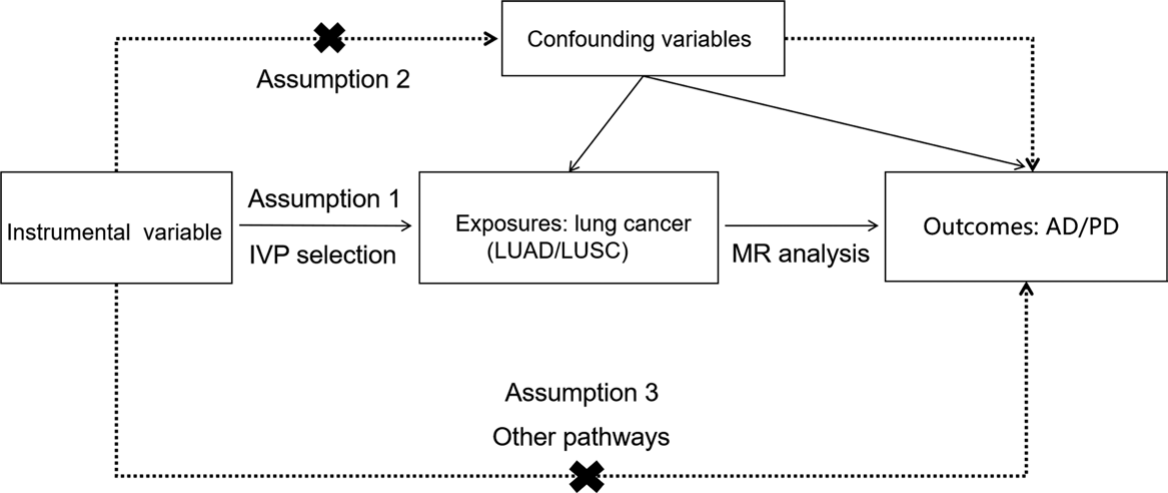
Assumptions of Mendelian randomization. The main assumptions form the basis of the Mendelian randomization: (1) relevance assumption; (2) independence assumption; (3) exclusive assumption.

In this study, we carried out MR analyses using a pooled dataset from the Genome-Wide Association Study (GWAS) database, to investigate whether exposures have a causal relationship (lung cancer, e.g., LUAD, LUSC) and outcomes (AD and PD). In addition, sensitivity analyses and pleiotropic tests were performed to verify the reliability of these findings. Furthermore, the basic principle of these tests is to use exposure-related IVs to evaluate the relationship between exposures and outcomes. Three core assumptions of MR studies included: relevance (IVs are closely associated with exposures); independence (IVs cannot be associated with any “exposure-outcome” related confounding variables); and exclusive assumptions (IVs can only affect outcomes through exposures). The schematic diagram is detailed in Figure 1. The guideline this study followed was the STROBE-MR guidance.^[13]^

### 2.2. Data source

In this study, the exposures (lung cancer, e.g., LUAD, LUSC) were obtained from the Transdisciplinary Research in Cancer of the Lung dataset. The LUAD dataset comprised 65,864 individuals, of whom 11,245 were patients with LUAD and 54,619 were healthy controls. The LUSC dataset consisted of 62,467 individuals, including 7704 patients with LUSC and 54,763 European healthy controls. In terms of outcomes, data on AD were derived from the dataset compiled by Bellenguez et al^[14]^ (with 39,106 patients with AD and 46,828 healthy controls), while data on PD were derived from the dataset compiled by Sakaue et al^[15]^ (with 2638 patients with PD and 477,380 healthy controls). Specific attributes of the datasets are displayed in Table 1. Meanwhile, we screened datasets of SCLC. However, they were excluded from the further research due to an insufficient number of IVs. The GWAS database included datasets analyzed in this study.

**Table 1:** Summary of the GWAS included in this MR study neoplasms.

Exposures/outcomes	GWASID	Consortium	Ethnicity	Sample sizes	Number of SNPs	Sex	Year
Lung enocarcinoma	ieu-a-984	TRIC	European	65,864	10,345,176	Males and females	NA
Squamous cell lung cancer	ieu-a-989	TRIC	European	62,467	103,415,291	Males and females	NA
AD	ebi-a-GCST90027158	Bellenguez et al^[14]^	European	487,511	20,921,626	NA	2022
PD	ebi-a-GCST90018894	Sakaue et al^[15]^	European	480,018	24,194,622	NA	2021

AD = Alzheimer disease, GWAS = Genome-Wide Association Studies, NA = not available, PD = Parkinson disease, SNPs = single nucleotide polymorphisms, TRIC = Transdisciplinary Research in Cancer of the Lung.

### 2.3. Selection and evaluation of IVs

SNPs that showed a significant correlation with exposures at the genome-wide level were selected, and the following parameters were set to remove the interference on linkage disequilibrium (LD): *P* < 5 × 10^−8^, *r*^2^ < 0.001, and genetic distance = 10,000 KB. Furthermore, the LDlink dataset (https://ldlink.nci.nih.gov/?tab=home) was utilized to ensure SNPs are not related to known confounding factors. The weak instrument bias was prevented by using the *F* statistic, which is calculated as follows:


$$\begin{array}{l} F=\left(N-k-1\right)/k\times \left[R^{2}/\left(1-R^{2}\right)\right] \end{array}$$

where N indicates the sample size in the GWAS analysis; *k* indicates the number of IV(s); *R*² indicates how much of the exposure is explained by the IV. Here’s how *R*² is specifically calculated:


$$\begin{array}{l} R^{2}=2\times \left(1-MAF\right)\times MAF\times \beta^{2} \end{array}$$

where *MAF* represents minor allele frequency; β represents the effect sizes of the SNP on the exposure. To see if the selected IVs had any weak instrumental bias, the *F* statistic was determined. All instruments are not weak if *F* > 10.

### 2.4. Data analysis

In this study, 5 MR techniques were used: IVW was the main technique, followed by weighted median, MR-Egger, simple mode, and weighted mode. In particular, for each IV in the causal estimate, Wald ratio estimates are combined to perform IVW. A random-effects model will be applied if there is heterogeneity, otherwise, a fixed-effects model will be employed. We also conducted sensitivity analyses on positive results from the MR analysis to make sure the results were accurate.

### 2.5. Quality control and visual analysis

The MR results were subjected to quality control using the following methods in order to confirm their reliability: First, the variation in the causal estimates found for every SNP is known as heterogeneity (i.e., the consistency of causal estimates for all SNPs, where reduced heterogeneity reflects higher accuracy in MR analysis findings). To determine whether there was heterogeneity, we used the Cochran *Q* test. If the *P*-value was higher than .05, there was no heterogeneity. Second, the horizontal pleiotropy analysis was performed with the MR-Egger intercept test. To be more specific, horizontal pleiotropy for genetic variants reflects the potential for a gene or genetic variant to have multiple independent phenotypic effects. If there is horizontal pleiotropy in the results, which was considered unreliable, and horizontal pleiotropy was deemed absent when *P* > .05. Third, the Mendelian randomization pleiotrophy residual sum and outlier test was adopted to discover outlier SNPs in the findings. If any outlier SNPs were found, they were removed from analysis and reanalyzed. Moreover, funnel plots were used to determine whether there was heterogeneity in the results. Fourth, the leave-one-out validation is an approach to measure the meta-effect of the remaining SNPs after removing each SNP individually. Then, we identified one or more SNPs that may have a substantial influence on the results by observing whether the results changed after each SNP was removed. Fifth, the results of MR were intuitively displayed in the forest plots. Sixth, scatter plots were used to evaluate the positive and negative linear relationships between exposures and outcomes.

### 2.6. Ethics statement

The study did not involve human participants and did not require ethical approval.

## 3. Results

### 3.1. MR analysis results with LUSC as an exposure

#### 3.1.1. Determination of IV and weak instrumental bias

We collected 9 SNPs related to LUSC from the GWAS database (*r*^2^ < 0.001, *P* < 5 * 10^–8^). If the outcome was AD, a total of 6 eligible SNPs remained after the alignment of alleles and elimination of palindromic sequences. If the outcome was PD, a total of 8 eligible SNPs remained after aligning the alleles and eliminating palindromic sequences. Every SNP had an *F* statistic larger than 10 (29.852–174.91), suggesting a lower possibility of weak instrumental bias.

#### 3.1.2. MR analysis results of LUSC and AD

**Figure 2.**
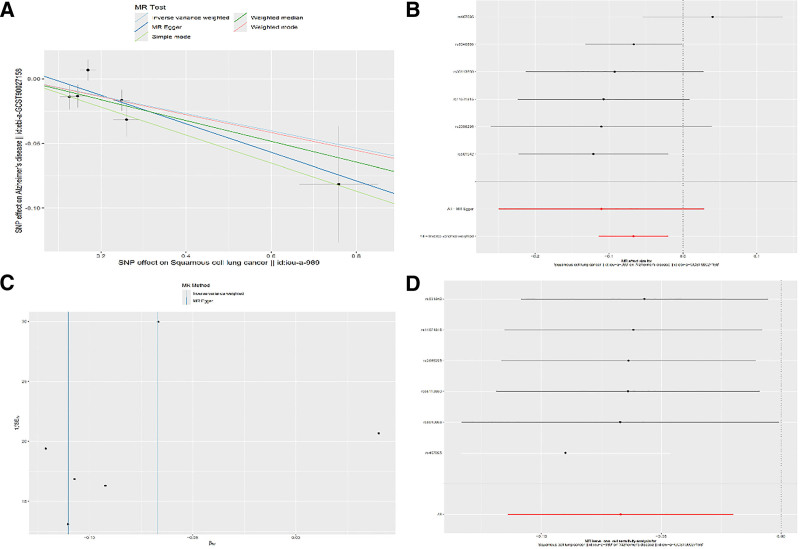
Visualization of MR results between LUSC and AD. (A) Scatter plot; (B) forest plot; (C) funnel plot; (D) leave-one-out validation. AD = Alzheimer disease, MR = Mendelian randomization.

We selected the IVW as the primary reference method, and findings manifested that LUSC and AD had a substantial negative connection (OR = 0.935, 95% CI [0.892–0.980], *P* = .005), which is detailed in Table 2. Additionally, the scatter plot displays the MR analysis results Figure 2A. The forest plots summarized the contribution of each SNP to the overall result, as displayed in Figure 2B. LUAD and AD were found to have a negative connection in the data above. In terms of sensitivity analysis, results of the MR-Egger intercept indicated that no pleiotropy was found at the gene level in this study (*P* = .548), as illustrated in Table 3, indicating our findings were reliable. The Cochran *Q* test results demonstrated that there was no evidence of heterogeneity in the findings (*P* = .223), which is detailed in Table 3. Similarly, the results of funnel plots indicated that there was no evidence of heterogeneity in the findings, as displayed in Figure 2C. The outcome of the leave-one-out validation is demonstrated in Figure 2D. According to these results, in the two-sample MR analyses, neither a single SNP nor a combination of special SNPs will significantly affect the outcome, indicating there was no bias or outliers. In conclusion, our findings are reliable.

**Table 2:** Mendelian randomization for LUSC and the risk of PD and AD.

Method	AD		Beta	PD		Beta
	OR estimate (95% CI)	*P*-value		OR estimate (95% CI)	*P*-value	
MR Egger	0.896 (0.779–1.029)	.194	−0.110	1.057 (0.675–1.662)	.813	0.057
Weighted median	0.923 (0.878–0.969)	.001	−0.081	0.856 (0.722–1.016)	.076	−0.155
Inverse variance weighted	0.935 (0.892–0.980)	.005	−0.067	0.920 (0.778–1.809)	.332	−0.083
Simple mode	0.897 (0.828–0.972)	.045	−0.109	0.802 (0.607–1.059)	.164	−0.221
Weighted mode	0.933 (0.875–0.995)	.089	−0.069	0.808 (0.674–0.969)	.055	−0.213

AD = Alzheimer disease, LUSC = lung squamous cell carcinoma, MR = Mendelian randomization, PD = Parkinson disease.

**Table 3:** Tests for heterogeneity and horizontal pleiotropy between LUSC and AD.

	Pleiotropy			Heterogeneity					
	MR-Egger			MR-Egger			Inverse variance weighted		
	Intercept	SE	*P*	*Q*	*Q*-df	*Q*-pval	*Q*	*Q*-df	*Q*-pval
AD	0.009	0.014	.548	6.292	4	0.178	0.698	5	0.223

AD = Alzheimer disease, LUSC = lung squamous cell carcinoma, MR = Mendelian randomization.

#### 3.1.3. MR analysis results of LUSC and PD

Five MR methods were employed to analyze the association between LUSC and PD. Among them, results of IVW revealed that there was no obvious connection between LUSC and PD (OR 0.920, 95%CI [0.778–1.089], *P* = .332 > 0.05). The remaining 4 MR analyses also suggested that MR results between LUSC and PD were not significant (*P* > .05), as shown in Table 2.

### 3.2. MR analysis results with LUAD as an exposure

#### 3.2.1. Determination of IV and weak instrumental bias

We extracted 14 SNPs related to LUAD from the GWAS database (*r*^2^ < 0.001, *P* < 5 * 10^–8^). If the outcome was AD, a total of 10 eligible SNPs were retained after aligning the alleles and eliminating palindromic sequences. If the outcome was PD, a total of 10 eligible SNPs remained after aligning the alleles and eliminating palindromic sequences. Every SNP had an *F* statistic larger than 10 (31.293–199.360), suggesting a lower possibility of weak instrumental bias.

#### 3.2.2. MR analysis results of LUAD and AD

To examine the correlation between the LUAD and AD, we employed 5 MR techniques: IVW, weighted median, MR-Egger, simple mode, and weighted mode. According to the results of IVW, LUAD did not negatively correlate with AD (OR = 0.968, 95% CI [0.915–1.025], *P* = .270 > .05). Conversely, the conclusions drawn from the weighted method revealed that LUAD may be negatively correlated with AD (OR = 0.916, 95% CI [0.848–0.988], *P* < .05). The remaining 3 MR analyses revealed no significant correlation between LUAD and AD (*P* > .05), as depicted in Table 4.

**Table 4:** Mendelian randomization for lung enocarcinoma on the risk of PD and AD.

Method	AD		Beta	PD		Beta
	OR estimate (95% CI)	*P*-value		OR estimate (95% CI)	*P*-value	
MR Egger	0.986 (0.802–1.213)	.898	−0.014	0.886 (0.671–1.170)	.418	−0.121
Weighted median	0.943 (0.886–1.003)	.062	−0.059	0.960 (0.814–1.131)	.622	−0.041
Inverse variance weighted	0.968 (0.915–1.025)	.270	−0.032	0.981 (0.851–1.130)	.787	−0.200
Simple mode	0.910 (0.791–1.047)	.220	−0.094	0.966 (0.748–1.249)	.800	−0.034
Weighted mode	0.916 (0.848–0.988)	.050	−0.088	0.932 (0.780–1.114)	.457	−0.071

AD = Alzheimer disease, MR = Mendelian randomization, PD = Parkinson disease.

#### 3.2.3. MR analysis results of LUAD and PD

The association between the LUAD and PD was examined using 5 different MR techniques. According to the results of IVW, MR results between LUAD and PD were not meaningful (OR = 0.981, 95% CI [0.851–1.130], *P* = .787 > 0.05). Similarly, conclusions from the other 4 MR analyses manifested that MR results between LUAD and PD were not meaningful (*P* > .05), as detailed in Table 4.

## 4. Discussion

A large number of studies have concluded that^[16–19]^ AD and PD, 2 neurological disorders that are frequent in the elderly, are similar in terms of their clinical manifestations, illness features, and pathological causes. This study employed a two-sample MR design to investigate the underlying genetic causal relationship between lung cancer and AD/PD. The results of the MR analysis revealed that there was a negative correlation between LUSC and AD.This study employed a two-sample MR design to investigate the underlying gen. However, MR analysis results, mainly using IVW, indicated that the correlation between LUAD and AD was not significant. MR analysis concluded that PD and lung cancer did not appear to be causally related.

The relationship between AD and cancer has been examined using MR techniques in a study.^[20]^ By classifying cancer into 2 types: cancer in smokers and cancer in nonsmokers, it explored the association between several cancers and AD. However, it only summarized the associations between AD and different cancers and did not further elaborate on the direct causal connection between AD and lung cancer. As a result, in order to establish the causal link between AD and lung cancer, we used MR techniques.

This study concluded that lung cancer may be negatively associated with AD, which yielded similar results with previous studies on epidemiological investigations of AD and decreased risk of cancer development.^[21,22]^ This conclusion may be attributed to the abnormal cell behaviors of both diseases in terms of pathological features. Specifically, AD is mostly distinguished by progressive loss of neurons at the early stage,^[23]^ while cancer is mostly distinguished by an unregulated proliferation of cancer cells.^[24]^

In addition, in patients with AD, genetic alteration may reduce the chance of cancer. For instance, in contrast to cancer, neurons in AD can sustain damage and die even without genetic alteration.^[25]^ Studies have proved^[26,27]^ that cancer patients had downregulated expression of multiple tumor suppressor genes (e.g., one of the first discovered tumor suppressor genes p53), whereas patients with AD had elevated expression of these genes. At the level of signal pathways, the activation of Wnt signal pathway may be one reason why the chance of lung cancer is decreased in those with AD. Wnt proteins, the signaling molecules produced by the Wnt gene family, involve in cell proliferation and differentiation, and are strongly correlated with the development of cancers.^[28]^ The study^[29]^ has confirmed that the activated Wnt signal pathway in AD counteracts the toxic effects of amyloid-β, thereby exerting a protective effect against AD. Moreover, it was reported that^[27]^ levels of heat shock proteins were generally lower in patients with AD. However, in cancer cells, a large number of proteins folded more frequently, resulting in an upregulation of heat shock protein to adapt to the heat shock response induced by cancer cells. The active activation of the immune system may also contribute to the inverse comorbidity pattern observed between the lung cancer and AD, according to a recent study.^[10]^ This is because neuroinflammation in patients with AD can lead to activation of the immune system in lung tissue, and the active immune response plays a positive role in the clearance of lung cancer cells. Additionally, the study by Sánchez-Valle et al^[30]^ concluded that dysregulation of oxidative phosphorylation and mitochondrial metabolism in patients with AD may also be a reason for the observed inverse comorbidity pattern with lung cancer. In this study, we found that LUSC was adversely connected to the incidence of AD. Conversely, between LUAD and AD, there was no obvious negative correlation. This could be connected to the differences in histopathology and anatomic position of 2 different types of lung cancer. LUSC mainly originates from the bronchial mucosa above the pulmonary segments, with the central-type being more prevalent. However, LUAD predominantly arises from bronchial mucous glands and spreads along the surrounding alveolar walls, forming pulmonary nodules at the lung boundary, primarily in a peripheral location.^[31]^ Pathologically, LUAD is a tumor with adenoid differentiation or mucin production from cancer cells, while LUSC is an epithelial tumor with keratinization and intercellular bridges. Additionally, most patients with LUAD are affected by environmental pollution, while most patients with LUSC are smokers.^[32]^ Notably, although earlier research revealed that AD might be related to the risk of lung cancer, which did not imply that there was a direct causal relationship between the 2, as patients with lung cancer normally have a lower survival rate, while the onset of patients with AD is often closely related aging. Furthermore, the conclusion of current studies on the link between AD and cancer could be biased due to interfering factors,^[33,34]^ including limited sample size, short follow-up periods, and inaccurate diagnosis. Given all this, future research is needed to better understand the mechanisms behind the prevalence of AD and lung cancer.

In earlier studies, some researchers believed that PD may be correlated with a lower incidence of cancer. For example, in a meta-analysis on the link between PD and cancer^[35]^ in >17 million patients with PD, a total of 63 articles were finally included. Among the 21 studies on smoking-related malignancies (such as lung, oral, and pharyngolaryngeal tumors), the pooled value of PD and related cancers was 0.76 (RR = 0.76, 95% CI [0.674–0.85], *P* < .001), indicating PD may be negatively correlated with smoking-related cancers. A cohort study conducted in Taiwan, China involved 4957 patients with PD, and findings revealed, compared with patients without PD, those with PD were less likely to acquire cancer than those without PD (HR = 0.88, 95% CI [0.78–0.99], *P* < .05), suggesting PD may be linked to reduced cancer incidence. Overall, PD and lower cancer incidence may be related for a variety of reasons. First, there are notable differences in the pathophysiology of these 2 diseases. The major feature of PD is the gradual loss of dopaminergic neurons, while cancer is mostly distinguished by unlimited cell proliferation and abnormal programmed cell death. In fact, progressive apoptosis in PD may inhibit the growth of cancer cells.^[7]^ Second, the treatment of PD patients requires the exogenous supplementation of levodopa. Studies have^[36]^ proved that dopamine is an effective treatment for malignant angiogenesis. Third, it was reported that^[37]^ dopamine D2 receptor (D2R) antagonists have anticancer efficacy in cell culture, which may be explained by how D2R antagonists inhibit the growth of cancer cells, inducing autophagy and apoptosis. As a result, PD may regulate apoptosis through D2R. Additionally, the mutation in the Parkin gene (PARK2) was found to be related to the anticancer effect of PD.^[38,39]^ Mutations in PARK2 are a common cause of PD, but increasing evidence suggested^[40,41]^ that the Parkin gene may act as a tumor suppressor. Moreover, PD and a reduced incidence of lung cancer were correlated, which may be ascribed to a decreased smoking rate in patients with PD. One of the main risk factors for lung cancer is smoking, but patients with PD are often affected by movement disorders, which can lead to a noticeable decrease in their smoking rates. Despite several links, the connection between PD and cancer is still controversial.

An American cohort study on the correlation between cancer and PD in older adults^[42]^ manifested that although the hazard ratio of PD in a single site (e.g., lung) was 0.81 (HR = 0.81, 95% CI [0.72–0.92], *P* < .05), when all cancer types and subsequent PD were combined (HR = 0.97; 95% CI [0.92–1.01], *P* > .05), cancer and PD were not shown to be related. Similarly, meta-analyses and MR methods were used by researchers to analyze the connection between prostate cancer and PD risk.^[43]^ According to the results of meta-analysis (RR = 0.89, 95% CI [0.73–1.08], *P* = .237) and MR analysis (OR = 1.025, 95%CI [0.997–1.054], *P* = .082), no notable association was found between these 2 conditions. Thus, prostate cancer and PD did not appear to be significantly correlated. In this MR study, no significant relationship was found between lung cancer and PD. Here are some potential explanations: First, PD is an environmentally-linked disorder. Recent evidence^[30]^ indicates that air pollution is major contributor to noncommunicable diseases worldwide. Long-term exposure to particulate matter may contribute to the development of both lung cancer and PD. Second, patients with PD are associated with activation of the PI3K-AKT-mTOR signaling pathway. The activation of this pathway affects the survival of dopaminergic neurons. Notably, this same signaling pathway is also implicated in tumor growth and invasion.^[44]^ Third, the pathogenesis of PD is linked to oxidative stress.^[45]^ Oxidative stress, through a series of pathways and processes such as mitochondrial stress, endoplasmic reticulum stress, and ferroptosis, also involves in the development of lung cancer.^[46]^ Additionally, the observed association may be partly related to the specificity of MR methods. The MR analysis tends to investigate the relationship between different diseases from a genetic perspective, potentially ignoring the influence of acquired factors. Further clinical and laboratory evidence is required to identify whether PD is associated with cancer.

This study evaluated the causal relationship between lung cancer and both AD and PD using the MR method, with the genetic variations of SNP as its IV, minimizing the interference of confounding factors presented in conventional observational studies. However, this study has some limits as well. First, every SNP was developed using data from the European population. Thus, the findings might not always apply to populations in different regions. Additionally, subgroup analyses based on the patient’s age, gender, and other potential confounding factors were not possible due to the use of GWAS data, which might introduce some heterogeneity in the results. Finally, confounding factors could not be fully controlled for in this investigation. In this study, following the elimination of SNPs associated with LD, the causality between SCLC and AD or PD remains unclear due to the limited number of SNPs. Therefore, numerous biological studies are still required in the future to further clarify the correlation between lung cancer and both AD and PD, reveal the biological mechanism underlying their negative correlation through in vivo and in vitro studies and further explore the common physiopathologic mechanisms between these 2 conditions, providing a solid theoretical basis for the creation of anticancer drugs based on the pathogenesis of neurodegenerative diseases.

## 5. Conclusion

In summary, we carried out two-sample MR analyses to analyze the potential genetic correlation between lung cancer and PD and AD. Our findings implied that lung cancer may be associated with a decreased incidence of AD.

## Author contributions

**Conceptualization:** Wentao Zheng, Ying Wang.

**Formal analysis:** Wentao Zheng, Ying Wang, Enjiang Zhu, Rui Xu, Ling Zhu.

**Investigation:** Wentao Zheng, Ying Wang, Enjiang Zhu, Rui Xu, Ling Zhu.

**Methodology:** Ying Wang.

**Resources:** Ying Wang.

**Supervision:** Ying Wang.

**Writing – original draft:** Wentao Zheng.

**Writing – review & editing:** Ying Wang, Enjiang Zhu, Rui Xu, Ling Zhu.
